# Auditory Frequency and Intensity Discrimination Explained Using a Cortical Population Rate Code

**DOI:** 10.1371/journal.pcbi.1003336

**Published:** 2013-11-14

**Authors:** Christophe Micheyl, Paul R. Schrater, Andrew J. Oxenham

**Affiliations:** 1 Department of Psychology, University of Minnesota, Minneapolis, Minnesota, United States of America; 2 Department of Computer Science, University of Minnesota, Minneapolis, Minnesota, United States of America; 3 Department of Otolaryngology, University of Minnesota, Minneapolis, Minnesota, United States of America; Newcastle University Medical School, United Kingdom

## Abstract

The nature of the neural codes for pitch and loudness, two basic auditory attributes, has been a key question in neuroscience for over century. A currently widespread view is that sound intensity (subjectively, loudness) is encoded in spike rates, whereas sound frequency (subjectively, pitch) is encoded in precise spike timing. Here, using information-theoretic analyses, we show that the spike rates of a population of virtual neural units with frequency-tuning and spike-count correlation characteristics similar to those measured in the primary auditory cortex of primates, contain sufficient statistical information to account for the smallest frequency-discrimination thresholds measured in human listeners. The same population, and the same spike-rate code, can also account for the intensity-discrimination thresholds of humans. These results demonstrate the viability of a unified rate-based cortical population code for both sound frequency (pitch) and sound intensity (loudness), and thus suggest a resolution to a long-standing puzzle in auditory neuroscience.

## Author Summary

A widely held view among auditory scientists is that the neural code for sound intensity (or loudness) involves temporally coarse spike-rate information, whereas the code for sound frequency (or pitch) requires more fine-grained and precise spike timing information. One problem with this view is that neurons in auditory cortex do not produce precisely time-locked responses to higher frequencies within the pitch range, suggesting that a transformation to a rate code must occur. However, because cortical neurons exhibit relatively broad tuning to frequency and correlated spike counts, it is unclear whether a cortical population code based on spike rates alone can support the remarkably precise pitch-discrimination ability of humans. Here we show that a relatively small population of virtual neurons with frequency-tuning and spike-count correlation characteristics consistent with those of actual neurons in the primary auditory cortex of primates, can account for both the smallest frequency- and intensity-discrimination thresholds measured behaviorally in humans. These results suggest a resolution to a long-standing puzzle in auditory neuroscience.

## Introduction

The nature of the neural code for perception is a fundamental question in neuroscience 1–5. In auditory neuroscience, the search for the neural code for pitch—an essential perceptual attribute of sound classes such as music and speech—has attracted considerable interest [6]–[9]. Two main types of neural codes for pitch have been offered: “timing” codes, which rely on fine spike-timing information [10], and “rate” codes, which involve spike rates computed over relatively long time windows—typically, a few hundred milliseconds [11].

Timing codes can carry considerably more information than rate codes [12], and the spike times of auditory-nerve fibers have been found to contain more information than needed to account for human listeners' ability to discriminate very small changes in frequency [11], [13], [14]. However, temporal coding degrades rapidly beyond the auditory nerve, making spike timing a less viable code at higher levels of neural processing. Indeed, in the primary auditory cortex, single units cannot precisely follow frequencies higher than a few hundred Hertz [15]–[17] – more than an order of magnitude below the upper limit of accurate pitch perception in humans [18]–[20]. Although studies in non-human animals found no deficits in pure-tone intensity or frequency discrimination following bilateral ablation of auditory cortex, substantial deficits in pure-tone frequency (pitch) and intensity (loudness) discrimination have been observed in human patients with cortical lesions [21], [22], suggesting that the auditory cortex plays an important role in those two perceptual abilities.

It seems likely, therefore, that any timing code for frequency in the auditory nerve is transformed into a cortical rate-place code. However, it is not known whether the information contained in the spike counts of a population of cortical neurons is sufficient to account for the very fine frequency-discrimination thresholds of human listeners. A cortical rate-place code for frequency discrimination faces two major obstacles: relatively broad receptive fields [23], implying poor resolution of small frequency differences by single units, and correlated spike counts [24], [25], which can severely limit the benefit of pooling information across multiple units [26]–[29].

Here, we examine the properties of a population of virtual neurons with frequency-tuning and spike-count correlation characteristics similar to those measured in the primary auditory cortex of primates. We determine that statistically optimal decoding of the information contained in the spike rates of these neurons can account quantitatively for the remarkable ability of trained human listeners to discriminate sound frequency. In addition, we show that the same cortical population code is also consistent with psychophysical data concerning another fundamental auditory ability: intensity discrimination. These results demonstrate the viability of a cortical rate code for both frequency and intensity discrimination, thus providing a possible resolution for a long-standing puzzle in auditory neuroscience.

## Results

**Figure 1:**
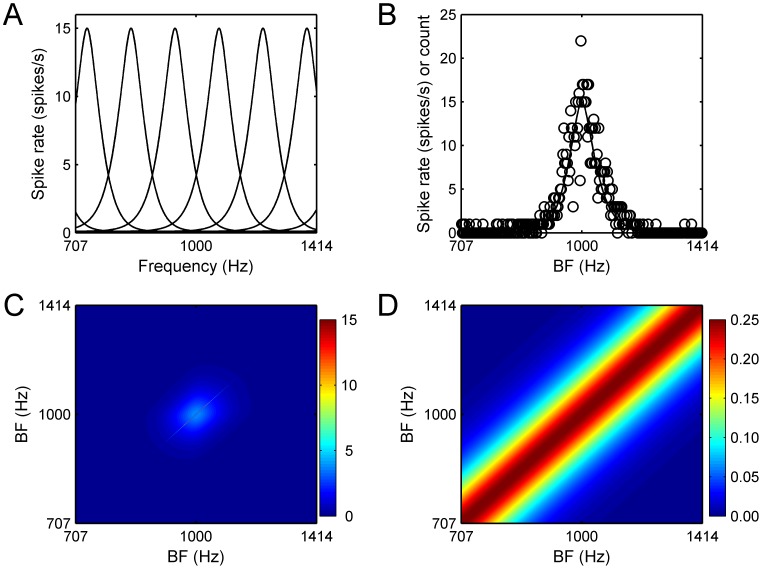
Example tuning curves, responses, and spike-count covariance matrix for the virtual cortical population. A. Example neural tuning curves for neurons with different BFs in primary auditory cortex. B. Example population response. The stimulus was a pure tone with a frequency of 1000: mean spike rates as a function of BF. Circles: simulated spike counts. C. Spike-rate covariance matrix. Entries on the diagonal correspond to spike-count variances for individual units and are equal to the spike rates shown in panel B. Off-diagonal entries correspond to covariances between the spike counts of different units, and are equal to the geometric mean of the units' spike rates times the spike-count correlation coefficient. D. Correlation matrix corresponding to the covariance matrix shown in panel C. See Methods for details.

Figure 1A shows frequency tuning curves (spike-rate versus stimulus frequency) for an array of virtual frequency-selective neurons with best frequencies (BFs) equally spaced on a logarithmic scale spanning a 1-octave range centered on 1 kHz. For illustration purposes, tuning curves are plotted for a small subset of units (*n* = 6) and a limited BF range, but the results described below are based on a larger number of units (*n* = 1700) and a wider BF range (2 octaves).

A key characteristic of neural tuning curves is their sharpness. A common measure of sharpness is the “quality factor” (*Q*), which is obtained by dividing the BF of the unit by a measure of tuning, in this case the width of the tuning curve at half of the peak spiking rate. The sharpness of the simulated units was adjusted to yield *Q* values consistent with those measured in the primary auditory cortex of primates, which have been found to equal 12 on average for sharply tuned units, and 3.7 on average for non-sharply tuned units [23]. Since sharp tuning is generally beneficial for frequency discrimination, in the context of this study we were interested primarily in discrimination performance based on the outputs of sharply tuned units. Thus, unless indicated otherwise, *Q* was set to 12. The tuning curves illustrated in Figure 1A reflect this choice.

Figure 1B shows simulated spike counts for this population of virtual neurons in response to a 1000 Hz, 50 dB SPL pure tone with a duration of 1 s. The spike counts were modeled as integer-valued random draws from a multivariate Gaussian distribution in which the variance of the spike counts for a given unit was equal to the unit's mean spike count—as is the case for Poisson-distributed spike counts. The covariance between the spike rates of two different units was either set to zero, reflecting an assumption of complete statistical independence between units, or to the product of the geometric mean spike rate and the spike-count correlation coefficient—consistent with the facts that 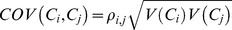, and 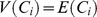 for all *i*, where *COV*(*C_i_*, *C_j_*), ρ*_i,j_*, *V*(*C_i_*), *V*(*C_j_*), *E*(*C_i_*), and *E*(*C_j_*) denote the covariance, correlation, variances, and expected values of the spike counts of units *i* and *j*, respectively. The latter covariance structure is consistent with neurophysiological data, which show decreasing spike-count correlations between pairs of cortical units as the distance between the units increases, and the overlap between their receptive fields decreases [24], [25], [30]–[32]. In the context of this article, the phrase “spike-count correlations” refers specifically to covariations in the spike counts of different units across multiple presentations of the same stimulus. Such correlations, also known as “noise correlations,” should not be confused with correlations between the spike counts of different units across different stimuli, which are traditionally referred to as “signal correlations” [33].

The resulting covariance and correlation matrices are shown in Figs. 1C and 1D, respectively. The correlation matrix was scaled so that the spike-count correlation coefficient (or, equivalently, the expected value of the correlation between the spike counts) of two units, ρ*_i,j_*, where *i* and *j* indicate different units, was maximally equal to ρ. Unless indicated otherwise, ρ was set to 0.25. This value was chosen based on recent findings, which indicate that such a value is not atypical for proximal cortical neurons [32], especially for output layers [34]. Even though higher discrimination performance might be achieved based on the response of cortical input layers [34], we reasoned that the properties of output layers of the primary auditory cortex were more relevant than those of other cortical layers for predicting the discrimination performance for a read-out mechanism located beyond the primary auditory cortex.

**Figure 2:**
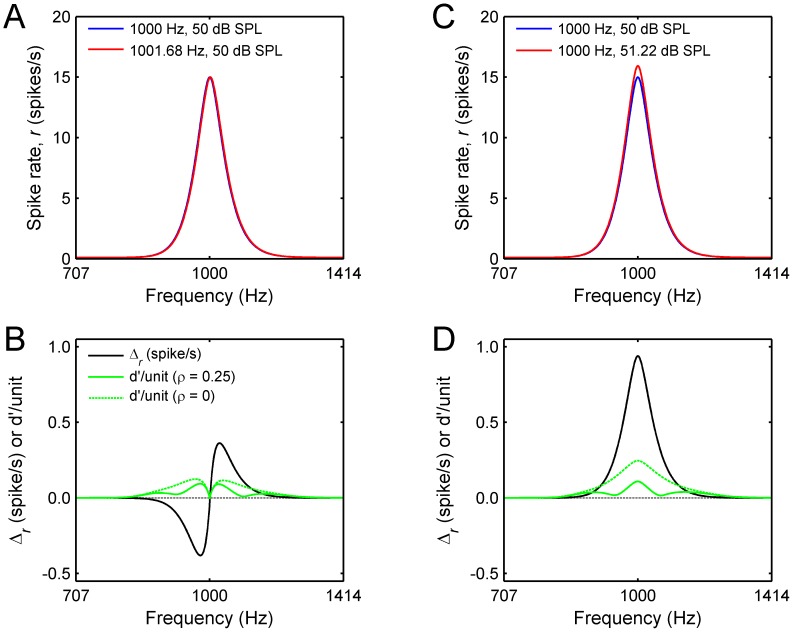
Population responses evoked by tones differing in frequency or intensity. A. Population responses evoked by two tones differing slightly in frequency. Blue: mean spike rate as function of BF for a 1000 Hz, 50 dB SPL pure tone. Red: mean spike rate as function of BF for a 1001.68 Hz, 50 dB SPL pure tone. B. Spike-rate difference (Δ*_r_*) and *d′*/unit as a function of BF. The spike-rate difference was obtained by subtracting the mean spike rate evoked by the higher-frequency stimulus (red curve in panel A) from the spike rate evoked by the lower-frequency stimulus (blue curve). *d′*/unit was computed as described in Experimental Procedures. C. Population responses evoked by two tones differing slightly in intensity. Blue: mean spike rate as function of BF for a 1000 Hz, 50 dB SPL pure tone. Red: mean spike rate as function of BF for 1000 Hz, 51.22 dB SPL pure tone. D. As for panel B, but for the population responses shown in panel C.

Figure 2A shows mean population responses evoked by two sequentially presented pure tones with slightly different frequencies: 1000 and 1001.68 Hz. The frequency difference, 1.68 Hz, corresponds approximately to the mean frequency-discrimination threshold (corresponding to a *d′* of 1) at 1000 Hz [35]. Note that the difference between the spike rates (*r*) evoked by the two tones (Figure 2B, black curve) is quite small relative to the variability of the spike counts (Figure 1B): across the entire population of neurons (*n* = 1700), the largest single-unit signal-to-noise ratio (SNR)—computed as the difference in spike rates evoked by the two stimuli (Δ*_r_*) divided by the square root of the spike rate evoked by the first stimulus [5]—was equal to 0.12. An SNR of 0.12 corresponds approximately to only 53% correct in a two-interval two-alternative forced-choice (2I2AFC) discrimination task [36], where chance performance is 50% correct. This leads to the question of how many units an optimal observer must pool spike-count information from in order to obtain the same performance as trained human listeners in this task, and with these stimuli, i.e., a *d′* of 1, or 76% correct in a 2I2AFC experiment. For statistically independent units with a constant spike-count covariance matrix, 
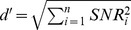
, where *SNR_i_* is the SNR (as defined above) for unit *i*. Therefore, if all the units in the population had uncorrelated spike counts and the same BF and tuning curve as the most informative unit (i.e., the unit for which SNR was the highest), combining spike-count information from ∼70 units would be sufficient to obtain a *d′* of 1.

In the presence of spike-count correlations, and of a stimulus-dependent covariance matrix, the relationship between overall performance (*d′*) and the single-unit SNRs (*SNR_i_*) is more complex, but *d′* can still be evaluated based on the Fisher information (see Methods) [37]. The Fisher information is inversely related to the Cramér-Rao lower bound on the variance of an estimator, which places a limit on the precision with which a quantity can be estimated using any decoding scheme (linear or nonlinear) [38], [39]; it is often used to quantify the best decoding performance that can be achieved based on the information contained in the responses of a population of neurons [27]–[29], [37]. Using this approach, we found that, for a population of units with tuning curves and spike-count correlations as illustrated in Figure 1, a *d′* of 1 was reached when the number of units in the population (with BFs spread evenly across the two-octave BF range), was set to 1700, which corresponds to a density of 850 units/octave. With no spike-count correlation (ρ = 0), a density of 300 units/octave was sufficient to obtain a *d′* of 1.0. Even if only 25% of units in primary auditory cortex are sharply tuned [23], a density of 850 sharply tuned units per octave implies an overall neuronal density of 3400 units per octave; this number is well within the range of physiologically realistic neuronal densities for the output layer of primary auditory cortex [40].

To gain insight into the effective contribution of each unit in the population to the overall performance, we computed the product of the square-root of the Fisher information for each unit and the frequency difference between the two stimuli (1.68 Hz), and plotted the resulting measure, *d′*/unit, as a function of BF. This was done for ρ = 0.25 (Figure 2B, solid green curve) and for ρ = 0 (Figure 2B, dashed green curve). Units with BFs more than ½ octave below, or above, the reference stimulus frequency (1 kHz) contributed very little to the overall discrimination performance. In fact, for ρ = 0.25, only 130 (∼8% of the 1700) units had a *d′*/unit larger than half of the *d′*/unit of the “best” (i.e., most informative) unit. Almost all of these units had BFs located within a frequency range of 2 semitones (12%, or 1/6^th^ of an octave) centered on the reference-stimulus frequency (1 kHz).

Our finding that a larger pool size is needed to reach the same performance (*d′* = 1) in the presence than in the absence of spike-count correlations is consistent with previous findings [26]. A simple explanation for the detrimental impact of spike-count correlations on stimulus discrimination performance is that they limit an observer's ability to “average out” neural noise without simultaneously canceling the signal. The “cost” of spike-count correlations on discrimination performance is apparent in the difference between the areas under the solid and dashed green curves (Figure 2B)—the square root of the sum of the squared *d′*/unit values across all units, which is equal to *d′*, was ∼70% larger for ρ = 0 than for ρ = 0.25. The “dip” at 1 kHz in the *d′*/unit curves stems from the fact that, for units with a BF close to 1 kHz, the difference between the spike rates evoked by the two stimuli (Δ*_r_*, black curve) was close to zero. The other dips, which are apparent in the dashed green curve, reflect the combined influence of the two factors that determine the Fisher information for each unit, namely, the change in spike rate (Δ*_r_*) and the change in the spike-count covariance matrix (see Methods). Intuitively, *d′*/unit values close to zero indicate units whose spike counts convey little information beyond that already provided by other units, once spike-count correlations are taken into account.

Figure 2C shows the mean population responses evoked by two tones having the same frequency (1000 Hz), but a different intensity (50 dB SPL versus 51.22 dB SPL). The intensity difference between the two stimuli (1.22 dB) was selected to represent the difference that corresponds to human discrimination sensitivity *d′* of 1, based on data in the psychoacoustic literature [41]. We determined the change in spike rate needed to obtain a *d′* of 1 using the same correlation coefficient (ρ = 0.25) and pool size (*n* = 1700), which were found earlier to yield a *d′* of 1 for the frequency-discrimination task. We found that a change in spike rate of slightly less than 1 spike/s (namely, 0.94 spikes/s) was sufficient. A spike-rate change of 0.94 spikes/s for a 1.22 dB change in sound intensity translates to a change of approximately 15 spikes/s for a 20-dB change in intensity. This value is consistent with example rate-level functions for neurons in primary auditory cortex in the literature, which typically show increases of 10 to 20 spike/s as the intensity of a tone at BF increases from 40 to 60 dB SPL [42]. Thus, it is possible to account for performance in the intensity-discrimination task using the same pool of sharply tuned units as assumed for the frequency-discrimination task with the same coarse rate-based neural code.

**Figure 3:**
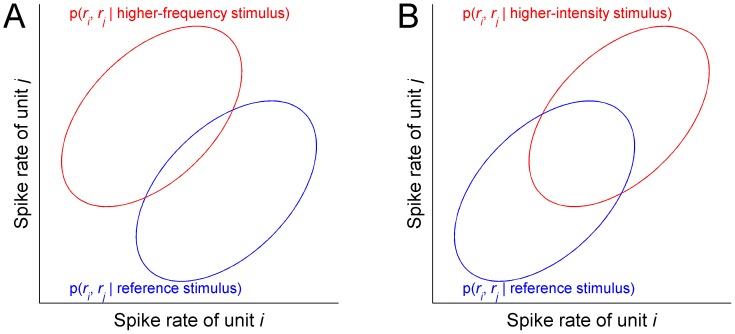
Schematic illustration of the impact of correlations on spike-rate distributions for frequency and intensity discrimination. A. Horizontal slices across spike-rate distributions evoked by two stimuli differing in frequency in two units with BFs below and above the reference-stimulus frequency. The spike rate of the unit (*i*) with BF lower than the reference frequency is plotted along the x-axis; the spike rate of the unit (*j*) with BF higher than the reference frequency is plotted along the y-axis. Blue: horizontal slice across the spike-rate distribution for the reference tone (1000 Hz, 50 dB SPL); red: horizontal slice across the spike-rate distribution for the higher-frequency tone. B. Same as A, but for an intensity change.

Note that the maximal change in spike rate (across all units) corresponding to the discrimination threshold was larger (by a factor of 2.5) for the intensity-discrimination task than for the frequency-discrimination task—compare the heights of the black curves in Figs. 2B and 2D. This outcome underscores the fact that equally discriminable stimulus differences need not correspond to equal differences in spike rates. It can be understood by considering the impact of spike-count correlations on the discrimination of frequency or intensity changes for a population containing only two units. Figure 3 shows equal-probability contours of probability distributions for spike counts (or single-trial estimates of spike-rates) evoked by tones differing in frequency (Figure 3A) or in intensity (Figure 3B), for two units, *i* and *j*. In this example, unit *i*, whose spike rates are plotted on the x-axis, has a BF below the reference stimulus frequency (1 kHz), while unit *j*, whose spike rates are plotted on the y-axis, has a BF above that frequency. When the frequency of the stimulus is increased, the spike rate of unit *i* decreases while that of unit *j* increases (Figure 3A). By contrast, when the intensity of the stimulus is increased, the spike rates of both units increase simultaneously (Figure 3B). Note that, for illustration purposes, the mean magnitude of the stimulus-induced changes in spike rate is the same for the two units and the two tasks. Under these circumstances, positive spike-count correlations, which are reflected in elongated contours along the major diagonal, lead to a smaller overlap between the two distributions for the frequency change than for the intensity change. Since the error rate of the optimal observer is directly related to the overlap between the spike-count probability distributions, for this case, positive correlations have a more dramatic impact on intensity-discrimination performance than on frequency-discrimination performance.

## Discussion

Historically, auditory researchers have found it difficult to account for both frequency discrimination and intensity discrimination within the same framework, or using the same neural code. This is in part because the changes in spike rate—or the changes in “excitation patterns” in psychoacoustical models— corresponding to threshold are usually smaller for a frequency-discrimination task than for an intensity-discrimination task [11], [13], [43], [44]. This has led to the view that intensity discrimination relies on spike-rate information whereas frequency discrimination requires fine spike-timing information—at least, for frequencies lower than about 8 kHz. One of the strongest arguments supporting this view stemmed from comparisons of discrimination thresholds measured in human listeners with predictions obtained using observer models that operate on spike-count information only, or use fine spike timing [13]. However, these models have traditionally been based on neural responses at the level of the auditory nerve, which contain a wealth of precise temporal information. The findings described above suggest a different conclusion at the level of the auditory cortex, where neurons are unable to accurately phase-lock to frequencies higher than, at most, a few hundred Hertz. We find that the spike rates of a realistically small population of units, with frequency-tuning and response-correlation characteristics similar to those observed in the primary auditory cortex of primates, contain enough statistical information to account for the smallest frequency-discrimination thresholds measured in human listeners—slightly less than 0.2%, or ∼2 Hz at 1 kHz.

Any viable rate-based population code for frequency (or pitch) discrimination must overcome two major limitations. The first limitation stems from the width of neural tuning curves: even the most sharply tuned units in the primary auditory cortex of primates have relatively wide receptive fields, with bandwidths (measured at half the peak spike-rate) of approximately 8% of the unit's best frequency (BF) [23]. One consequence of such wide receptive fields is that the change in spike rate produced by a small (e.g., 0.2%) change in stimulus frequency is very small relative to the neural noise, i.e., the random variability in spike counts. In principle, the detectability of small spike-rate differences can be enhanced by pooling information across many neural units. However, previous work in theoretical neuroscience has indicated that the benefit of pooling spike-count information across multiple units can be drastically limited if the pooled spike counts are correlated [26]–[29], [37]. The spike counts of cortical neurons are correlated [24]–[26], [30], [32]–[34]. Thus, it was unclear *a priori* whether a population code based solely on spike-rate information in auditory cortex could support the remarkably fine frequency-discrimination performance of humans. The results described above offer a positive answer to this question. They show that, contrary to popular belief, a cortical rate-place code can provide sufficient information to account for human behavior in the dimensions of both frequency and intensity, using reasonable assumptions relating to unit density, unit tuning, and inter-unit correlations.

As with any modeling study, the conclusions of this work depend on the assumptions of the underlying model. In particular, our estimates of the number of units needed to achieve a given level of behavioral discrimination performance rely on the assumption that downstream neurons use the information contained in the spike counts of the population optimally in a statistical (maximum-likelihood) sense. It remains to be determined whether neural networks in the auditory cortex can achieve, or even approach, this optimum. If they cannot, the estimated numbers of neurons needed to explain the behavioral performance of human listeners in the frequency- and intensity-discrimination task would be under-estimates. Importantly, however, increasing the assumed population size would not necessarily alter our main conclusion, according to which the behavioral thresholds for these two tasks can, at least in theory, be accounted for using the same population and same type of (spike-rate) code. Another assumption on which our conclusions may depend relates to the strength of spike-count correlations and its relationship with other characteristics, such as the BFs and frequency-tuning widths of the units. Our choice of correlation structure for the virtual population was based in part on neurophysiological data [24], [25], and in part on theoretical and simplicity considerations. Lastly, the conclusions of this study are subject to the limitations of Fisher information as a measure of optimum decoding performance for neural populations [e.g., 45].

Our finding that a cortical population code operating solely on spike-count information can account for frequency-discrimination performance in humans has important implications for the search of neural correlates of frequency (pitch) perception in humans. For example, while explanations for the dependence of frequency-discrimination thresholds on stimulus parameters such as frequency, intensity, and duration, have so far focused almost exclusively on peripheral (i.e., cochlear and auditory-nerve) response properties, our approach provides a method for examining the role of central factors, such as variations in neuronal density [46], [47] or in spike-count correlations across BFs at the cortical level, in determining behavioral discrimination thresholds.

## Methods

Responses of a population of frequency-selective cortical units were simulated as follows. The spike rate (in spikes/s) for unit *i* (*i* = 1,…, *n*) in response to a tone of frequency, *f*, and intensity, *l*, was computed as,
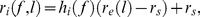
(1)where *r_e_*(*l*) and *r_s_* denote the stimulus-evoked spike rate at BF and the spontaneous spike rate, respectively, and *h_i_*(*f*) represents the frequency-tuning function,
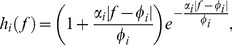
(2)in which φ*_i_* denotes the BF (in Hz) of unit *i*, and the sharpness parameter, α*_i_*, was adjusted to yield a quality factor, *Q*, consistent with that of single units in the primary auditory cortex of primates [23]. This function is sometimes referred to as the “rounded exponential” (*roex*) function, and has been used to model psychophysical auditory-filter shapes [48] as well as neural frequency-tuning curves in the primary auditory cortex of primates [49]. The spontaneous rate, *r_s_*, was set to 0.1 spikes/s and the evoked rate, *r_e_*, for a 50 dB SPL pure tone having a frequency equal to the BF of the unit was set to15 spikes/s. These numbers are consistent with neurophysiological data [42]. Other physiologically realistic values for these parameters (e.g., *r_s_* = 1 and *r_e_* = 10 or 20) were also tested and led to qualitatively similar conclusions.

Spike counts were simulated by drawing samples from a multivariate Gaussian probability density function with mean vector, ***r***(*f*, *l*) = [*r*_1_(*f*, *l*), …, *r_n_*(*f*, *l*)], and covariance matrix, **V**(*f*, *l*),
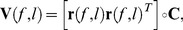
(3)where ○ denotes the Hadamard (entrywise) matrix product.

Consistent with neurophysiological data indicating that spike-count correlations for neuron pairs in primary auditory cortex tend to decrease with increasing BF distance and decreasing receptive-field overlap between the units [24], [25], the spike-rate correlation matrix, **C**, was defined as,
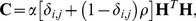
(4)where δ*_i_*_,*j*_ = 1 for *i* = *j* and δ*_i_*_,*j*_ = 0 for *i*≠*j*, and **H** = [***h***_1_(**φ**), …, ***h*** *_n_*(**φ**)], in which the elements of each *n*-vector, ***h*** *_i_*(**φ**), were equal to 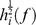 evaluated at *f* = **φ** *_j_*, *j* = 1,…, *n*, and 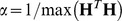. To obtain integer-valued spike counts, samples from the multivariate Gaussian probability density function were rounded to the nearest unit.

Neglecting the effect of rounding, the highest frequency-discrimination performance, *d′_f_*, that can be obtained using the information contained in the spike counts of a population of units with characteristics as described above can be determined as [42], [47],
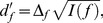
(5)where Δ*_f_* denotes the frequency difference between the two tones being discriminated, and *I*(*f*) denotes the Fisher information for frequency,
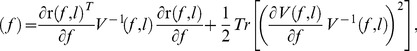
(6)where *Tr* denotes the trace operator. The partial derivative of the rate vector with respect to frequency, 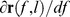, can be determined based on the preceding equations.

Analogous equations were used to compute performance for an intensity-discrimination task. To compute the partial derivative of the rate vector with respect to sound intensity, 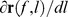, we assumed that the spike rate varied linearly with the stimulus intensity (in dB), and adjusted the constant of proportionality between these two variables to yield the desired performance (*d′* = 1) for the intensity-discrimination task.

Unit-specific measures of performance, *d′*/unit, were computed as,
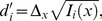
(7)with,
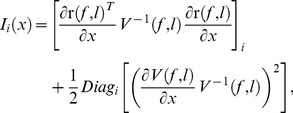
(8)where *x* can be either *f* (frequency) or *l* (intensity), [.]*_i_* denotes the *i* ^th^ element of its (vector) argument, and *Diag_i_*[.] denotes the *i* ^th^ element of the diagonal of its argument. The stimulus difference, Δ*_x_*, was set to the discrimination threshold (corresponding to *d′* = 1) for the considered task: 1.68 Hz for frequency discrimination, and 1.22 dB for intensity discrimination.

## References

[1] Bialek W, Rieke F, de Ruyter van Steveninck RR, Warland D (1991) Reading a neural code. Science 252: 1854–1857. 2063199 10.1126/science.2063199

[2] Eggermont JJ (2001) Between sound and perception: reviewing the search for a neural code. Hear Res 157: 1–42. 11470183 10.1016/s0378-5955(01)00259-3

[3] Jacobs AL, Fridman G, Douglas RM, Alam NM, Latham PE, et al (2009) Ruling out and ruling in neural codes. Proc Natl Acad Sci U S A 106: 5936–5941. 19297621 10.1073/pnas.0900573106 PMC2657589

[4] Parker AJ, Newsome WT (1998) Sense and the single neuron: probing the physiology of perception. Annu Rev Neurosci 21: 227–277. 9530497 10.1146/annurev.neuro.21.1.227

[5] Rieke F, Warland D, de Ruyter van Steveninck R, Bialek W (1997) Spikes: Exploring the Neural Code. Cambridge: MIT Press.

[6] Bendor D, Wang X (2005) The neuronal representation of pitch in primate auditory cortex. Nature 436: 1161–1165. 16121182 10.1038/nature03867 PMC1780171

[7] Griffiths TD (2012) Cortical mechanisms for pitch representation. J Neurosci 32: 13333–13334. 23015421 10.1523/JNEUROSCI.1661-12.2012 PMC6621357

[8] Oxenham AJ (2013) Pitch perception. J Neurosci 32: 13335–13338. 10.1523/JNEUROSCI.3815-12.2012 PMC3481156 23015422

[9] Walker KM, Bizley JK, King AJ, Schnupp JW (2011) Cortical encoding of pitch: recent results and open questions. Hear Res 271: 74–87. 20457240 10.1016/j.heares.2010.04.015 PMC3098378

[10] Cariani PA, Delgutte B (1996) Neural correlates of the pitch of complex tones. I. Pitch and pitch salience. J Neurophysiol 76: 1698–1716. 8890286 10.1152/jn.1996.76.3.1698

[11] Siebert WM (1970) Frequency discrimination in the auditory system: place or periodicity mechanisms? Proc IEEE 723–730.

[12] Borst A, Theunissen FE (1999) Information theory and neural coding. Nat Neurosci 2: 947–957. 10526332 10.1038/14731

[13] Heinz MG, Colburn HS, Carney LH (2001) Evaluating auditory performance limits: i. one-parameter discrimination using a computational model for the auditory nerve. Neural Comput 13: 2273–2316. 11570999 10.1162/089976601750541804

[14] Moore BCJ (1973) Frequency difference limens for short-duration tones. J Acoust Soc Am 54: 610–619. 4754385 10.1121/1.1913640

[15] De Ribaupierre F, Goldstein MH Jr, Yeni-Komshian G (1972) Intracellular study of the cat's primary auditory cortex. Brain Res 48: 185–204. 4345594 10.1016/0006-8993(72)90178-3

[16] Elhilali M, Fritz JB, Klein DJ, Simon JZ, Shamma SA (2004) Dynamics of precise spike timing in primary auditory cortex. J Neurosci 24: 1159–1172. 14762134 10.1523/JNEUROSCI.3825-03.2004 PMC6793586

[17] Wang X, Lu T, Bendor D, Bartlett E (2008) Neural coding of temporal information in auditory thalamus and cortex. Neuroscience 157: 484–494. 19143093 10.1016/j.neuroscience.2008.07.050

[18] Moore BC, Ernst SM (2012) Frequency difference limens at high frequencies: Evidence for a transition from a temporal to a place code. J Acoust Soc Am 132: 1542–1547. 22978883 10.1121/1.4739444

[19] Oxenham AJ, Micheyl C, Keebler MV, Loper A, Santurette S (2011) Pitch perception beyond the traditional existence region of pitch. Proc Natl Acad Sci U S A 108: 7629–7634. 21502495 10.1073/pnas.1015291108 PMC3088642

[20] Moore BCJ (2003) An Introduction to the Psychology of Hearing. London: Academic Press.

[21] Tramo MJ, Shah GD, Braida LD (2002) Functional role of auditory cortex in frequency processing and pitch perception. J Neurophysiol 87: 122–139. 11784735 10.1152/jn.00104.1999

[22] Dykstra AR, Koh CK, Braida LD, Tramo MJ (2012) Dissociation of detection and discrimination of pure tones following bilateral lesions of auditory cortex. PLoS One 7: e44602. 22957087 10.1371/journal.pone.0044602 PMC3434164

[23] Bartlett EL, Sadagopan S, Wang X (2011) Fine frequency tuning in monkey auditory cortex and thalamus. J Neurophysiol 106: 849–859. 21613589 10.1152/jn.00559.2010 PMC3154823

[24] Eggermont JJ (1992) Neural interaction in cat primary auditory cortex. Dependence on recording depth, electrode separation, and age. J Neurophysiol 68: 1216–1228. 1432079 10.1152/jn.1992.68.4.1216

[25] Brosch M, Schreiner CE (1999) Correlations between neural discharges are related to receptive field properties in cat primary auditory cortex. Eur J Neurosci 11: 3517–3530. 10564360 10.1046/j.1460-9568.1999.00770.x

[26] Zohary E, Shadlen MN, Newsome WT (1994) Correlated neuronal discharge rate and its implications for psychophysical performance. Nature 370: 140–143. 8022482 10.1038/370140a0

[27] Averbeck BB, Latham PE, Pouget A (2006) Neural correlations, population coding and computation. Nat Rev Neurosci 7: 358–366. 16760916 10.1038/nrn1888

[28] Averbeck BB, Lee D (2006) Effects of noise correlations on information encoding and decoding. J Neurophysiol 95: 3633–3644. 16554512 10.1152/jn.00919.2005

[29] Series P, Latham PE, Pouget A (2004) Tuning curve sharpening for orientation selectivity: coding efficiency and the impact of correlations. Nat Neurosci 7: 1129–1135. 15452579 10.1038/nn1321

[30] Bair W, Zohary E, Newsome WT (2001) Correlated firing in macaque visual area MT: time scales and relationship to behavior. J Neurosci 21: 1676–1697. 11222658 10.1523/JNEUROSCI.21-05-01676.2001 PMC6762960

[31] Ecker AS, Berens P, Keliris GA, Bethge M, Logothetis NK, et al (2010) Decorrelated neuronal firing in cortical microcircuits. Science 327: 584–587. 20110506 10.1126/science.1179867

[32] Smith MA, Kohn A (2008) Spatial and temporal scales of neuronal correlation in primary visual cortex. J Neurosci 28: 12591–12603. 19036953 10.1523/JNEUROSCI.2929-08.2008 PMC2656500

[33] Cohen MR, Kohn A (2011) Measuring and interpreting neuronal correlations. Nat Neurosci 14: 811–819. 21709677 10.1038/nn.2842 PMC3586814

[34] Hansen BJ, Chelaru MI, Dragoi V (2012) Correlated variability in laminar cortical circuits. Neuron 76: 590–602. 23141070 10.1016/j.neuron.2012.08.029 PMC3653617

[35] Micheyl C, Xiao L, Oxenham AJ (2012) Characterizing the dependence of pure-tone frequency difference limens on frequency, duration, and level. Hear Res 292: 1–13. 22841571 10.1016/j.heares.2012.07.004 PMC3455123

[36] Green DM, Swets JA (1966) Signal Detection Theory and Psychophysics. New York: Krieger.

[37] Abbott LF, Dayan P (1999) The effect of correlated variability on the accuracy of a population code. Neural Comput 11: 91–101. 9950724 10.1162/089976699300016827

[38] Kay SM (1993) Fundamentals of Statistical Signal Processing: Estimation Theory. Englewood Cliffs, NJ: Prentice-Hall.

[39] Dayan P, Abbott LF (2001) Theoretical Neuroscience. Computational and Mathematical Modeling of Neural Systems. Cambridge, MA: MIT Press.

[40] Collins CE, Airey DC, Young NA, Leitch DB, Kaas JH (2010) Neuron densities vary across and within cortical areas in primates. Proc Natl Acad Sci U S A 107: 15927–15932. 20798050 10.1073/pnas.1010356107 PMC2936588

[41] Jesteadt W, Wier CC, Green DM (1977) Intensity discrimination as a function of frequency and sensation level. J Acoust Soc Am 61: 169–177. 833368 10.1121/1.381278

[42] Calford MB, Semple MN (1995) Monaural inhibition in cat auditory cortex. J Neurophysiol 73: 1876–1891. 7623087 10.1152/jn.1995.73.5.1876

[43] Moore BC, Sek A (2009) Sensitivity of the human auditory system to temporal fine structure at high frequencies. J Acoust Soc Am 125: 3186–3193. 19425661 10.1121/1.3106525

[44] Moore BCJ, Glasberg BR (1989) Mechanisms underlying the frequency discrimination of pulsed tones and the detection of frequency modulation. J Acoust Soc Am 86: 1722–1732.

[45] Berens P, Ecker AS, Gerwinn S, Tolias AS, Bethge M (2011) Reassessing optimal neural population codes with neurometric functions. Proc Natl Acad Sci USA 108: 4423–4428. 21368193 10.1073/pnas.1015904108 PMC3060259

[46] Irvine DR, Rajan R (1996) Injury- and use-related plasticity in the primary sensory cortex of adult mammals: possible relationship to perceptual learning. Clin Exp Pharmacol Physiol 23: 939–947. 8911738 10.1111/j.1440-1681.1996.tb01146.x

[47] Plumbley MD (1999) Do cortical maps adapt to optimize information density? Network 10: 41–58. 10372761

[48] Patterson RD, Nimmo-Smith I (1980) Off-frequency listening and auditory filter asymmetry. J Acoust Soc Am 67: 229–245. 7354191 10.1121/1.383732

[49] Fishman YI, Micheyl C, Steinschneider M (2013) Neural representation of harmonic complex tones in primary auditory cortex of the awake monkey. J Neurosci 33: 10312–10323. 23785145 10.1523/JNEUROSCI.0020-13.2013 PMC3685833

